# 浦东新区不同组织学类型肺癌发病及生存情况

**DOI:** 10.3779/j.issn.1009-3419.2014.03.04

**Published:** 2014-03-20

**Authors:** 涵一 陈, 琛 杨, 蓓 闫, 良红 孙, 铮 吴, 小攀 李, 美玉 张, 晓莉 李, 黎明 杨

**Affiliations:** 200136 上海，上海市浦东新区疾病预防控制中心，肿瘤、伤害防治与生命统计科 Department of Cancer, Injury Prevention and Vital Statistics, Pudong New Area Center for Disease Control and Prevention, Shanghai 200136, China

**Keywords:** 肺肿瘤, 生存分析, 发病趋势, 影响因素, Lung neoplasms, Survival analysis, Incidence trend, Influential factors

## Abstract

**背景与目的:**

不同组织学类型肺癌患者发病特点、预后情况不同。本研究旨在分析不同组织学类型肺癌患者发病情况、发病趋势、生存期及其影响因素，为病因学研究和临床防治提供参考依据。

**方法:**

以2002年-2009年上海市浦东新区居民为研究对象，利用年度变化百分比进行肺癌发病趋势分析。通过寿命表法计算生存率，以*Log-rank*检验生存曲线差异。

**结果:**

该地区同期肺癌男女标化发病率分别为52.28/10万和18.86/10万，明确组织学分型的肺癌患者中位生存期为410.72天。腺癌发病率最高并成上升趋势（*P* < 0.05）。小细胞癌生存情况最差。原南汇地区男性鳞癌生存情况较好。

**结论:**

不同组织学类型肺癌发病趋势和生存期并不相同，性别、年龄、居住地对不同组织学类型肺癌患者生存期有影响。

## Abstract (en)

**Background and objective:**

Different histologies of lung cancer vary in occurrence and prognosis. This study aims to analyze the incidence and occurrence trend of lung cancer and investigate the survival rate and its influential factors among lung cancer patients with different histologies.

**Methods:**

Permanent residents were recruited between 2002 and 2009 in Pudong New Area (former Nanhui Area and former Pudong Area), Shanghai, China. Annual percent changes were estimated by a linear regression of the logarithm on the incidence rates for eight years. Survival rates were calculated and compared by using life-table analysis and *Log-rank* test, respectively.

**Results:**

The standardized incidence rates of lung cancer were 52.28 and 18.86 per 100, 000 in males and females, respectively. The median survival time was 410.72 days for specific classified lung cancer. The incidence rates of adenocarcinoma ranked the highest and showed an upward tendency (*P* < 0.05). Patients with small cell lung cancer showed the worst survival condition. The survival condition in males with squamous cell lung cancer living in former Nanhui Area was better compared with those living in former Pudong Area.

**Conclusion:**

Lung cancers with different histologies demonstrated different occurrence trends and survival rates. Gender, age, and living area influence the survival rates of lung cancer with different histologies.

肺癌是威胁人类健康的主要恶性肿瘤之一，全球每年新发肺癌病例约160万^[1]^。随着我国疾病谱的改变，肺癌已成为我国城乡恶性肿瘤死亡首要原因，占全部恶性肿瘤死亡的22.7%^[2]^。

不同组织学类型肺癌患者发病特点、治疗方式、预后情况明显不同^[3, 4]^。本研究利用浦东新区肿瘤登记报告和随访管理系统，分析该地区不同组织学类型肺癌发病情况以及患者生存情况，对2002年-2009年浦东新区不同组织学类型肺癌发病情况及发病趋势进行评估，探索影响不同组织学类型肺癌患者生存期的影响因素，为病因学研究和临床防治提供参考依据。

## 1 资料与方法

### 1.1 资料来源与研究方法

上海市浦东新区由原浦东与原南汇两区合并而来，前者处于上海龙头地位、城市化明显，而后者仍保留大量的农村地区。本研究以该地区常住户籍人口为研究对象。根据疾病国际分类第10版（ICD-10），纳入编码为C33、C34的支气管和肺恶性肿瘤患者，并以国际肿瘤学分类标准编码（ICD-O-3）进行分类。对于有多项诊断依据的患者，最高诊断依据以病理（包括细胞学、血片等）、生化/免疫、无病理的手术/尸检、X线/CT/内窥镜、以及临床诊断的顺序由高到低进行选择。根据患者家庭住址分为原南汇和原浦东两个地区。

### 1.2 统计学方法及研究指标

本研究应用STATA 11.0统计软件进行研究和统计分析。发病率的计算以浦东新区常住户籍人口为基础，各年平均人口数为该年年初与年末人口数的平均值，按不同肺癌组织学类型分别计算，采用WHO世界标准人口年龄构成进行标化^[5]^。年度变化百分比（annual percent change, APC）应用线性回归按病例数加权后进行率的计算和率值趋势检验，通过β系数计算而来^[6]^。寿命表法计算肺癌患者的生存率，以*Log-rank*检验不同分组的生存曲线差异，检验水准α=0.05。

## 2 结果

### 2.1 人口概述

浦东新区2009年底户籍人口数为270.44万，比2002年底的239.91万增加了逾30万人口，人口增长率为12.73%。2002年-2009年，65岁以上老年人群所占比例呈上升趋势，65岁以上人口年龄构成均已超过7%，达到12%以上。

### 2.2 发病概况

2002年-2009年，浦东新区共登记报告新发肺癌13, 417例，肺癌粗发病率（crude incidence rate, CIR）为65.59/10万，标化发病率（age-standardized rate, ASR）为34.42/10万。其中男性CIR、ASR分别为92.64/10万和52.28/10万，女性为38.48/10万和18.86/10万，男女标化发病性别比为2.77:1。男性肺癌粗发病率没有明显变化，女性则呈上升趋势，其APC值为2.708%（*t*=2.87, *P*=0.028）。但标化后，男性发病率呈下降趋势，其APC值为-2.817%（*t*=-3.44, *P*=0.014），女性发病率变化趋势则没有统计学意义。

### 2.3 不同组织学类型发病情况

2002年-2009年，浦东新区明确组织学分型的新发肺癌5, 648例，占同期肺癌发病者的42.1%。腺癌发病率最高，男女ASR分别为10.13/10万和5.81/10万；腺癌发病率呈明显上升趋势，男、女标化发病率APC值分别为3.777%（*t*=3.42, *P*=0.014）和5.753%（*t*=3.45, *P*=0.014）。鳞癌发病率次之，男女ASR分别为8.42/10万和2.02/10万；男性鳞癌发病率无明显变化趋势，而女性鳞癌粗发病率成上升趋势，APC值为6.037%（*t*=2.57, *P*=0.042）。男、女小细胞癌ASR分别为1.64/10万和0.48/10万，男、女腺鳞癌ASR分别为1.09/10万和0.35/10万；男性小细胞癌和腺鳞癌粗发病率均成上升趋势，但在女性则无上述变化，详见表 1和图 1。病理学/细胞学诊断是最主要的诊断依据，依据该诊断分型的主要组织学类型超过92%，但其仅占全部诊断依据的47.08%，见表 2。

**1:** 2002-2009年浦东新区居民肺癌发病情况及发病趋势 Incidence rates and trend of lung cancer in Pudong New Area, 2002-2009

Histologies	Gender	Case	CIR					ASR			
			Rate (1/100, 000)	APC (%)	*t*	*P*		Rate (1/100, 000)	APC (%)	*t*	*P*
Squamous cell carcinoma	M	1, 465	14.31	1.859	1.73	0.134		8.42	-2.103	-1.55	0.171
	F	402	3.94	6.037	2.57	0.042		2.02	3.215	1.33	0.233
Adenocarcinoma	M	1, 826	17.83	8.255	6.27	0.001		10.13	3.777	3.42	0.014
	F	1, 005	9.84	9.133	5.97	0.001		5.33	5.753	3.45	0.014
Adenosquamous carcinoma	M	185	1.81	8.532	2.47	0.048		1.09	4.870	1.77	0.127
	F	66	0.65	7.922	1.95	0.099		0.35	3.928	1.26	0.256
Small cell carcinoma	M	297	2.9	8.691	3.26	0.017		1.64	4.325	1.82	0.118
	F	95	0.93	7.836	1.73	0.134		0.48	1.587	0.32	0.760
Other specific lung cancer	M	223	2.18	-3.077	-0.54	0.610		1.26	-6.844	-1.16	0.290
	F	84	0.82	-1.024	0.23	0.826		0.46	1.876	0.41	0.695
Total	M	9, 486	92.64	1.300	1.92	0.103		52.28	-2.817	-3.44	0.014
	F	3, 931	38.48	2.708	2.87	0.028		18.86	-1.270	-1.66	0.148
CIR: crude incidence rate; ASR: age-standardized rate.											

**1:**
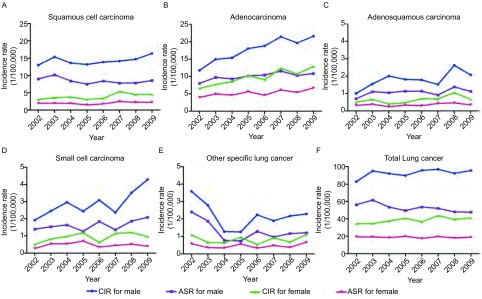
2002年-2009年上海市浦东新区不同组织学类型肺癌发病趋势。A-D：男、女鳞癌、腺癌、腺鳞癌、小细胞癌四种明确组织类型肺癌粗发病率和标化发病率随时间变化趋势；E：其他明确组织类型肺癌分性别粗发病率和标化发病率随时间变化趋势；F：分性别肺癌总体粗发病率和标化发病率随时间变化趋势。 The incidence trend of lung cancer with different histologies in Pudong New Area, Shanghai, 2002-2009. A-D:Time trend of crude incidence rate (CIR) and age-standardized rate (ASR) for male and female on squamous cell carcinoma, adenocarcinoma, adenosquamous carcinoma, small cell carcinoma; E: Time trend of CIR and ASR for male and female on other specific lung cancer; F: Time trend of CIR and ASR on total lung cancer for male and female.

**2:** 2002年-2009年浦东新区居民不同组织学类型肺癌最高诊断依据 The highest diagnostic basis of lung cancer by different histologies in Pudong New Area, 2002-2009

Diagnostic basis	Histologies case^*^						
	Squamous cell carcinoma	Adeno- carcinoma	Adenosquamous carcinoma	Small cell carcinoma	Others	No classification	Total
Clinic	15 (0.80%)	24 (0.85%)	1 (0.40%)	8 (2.04%)	8 (2.61%)	1, 763 (22.70%)	1, 819 (13.56%)
Iconography	91 (4.87%)	117 (4.13%)	5 (1.99%)	15 (3.83%)	61 (19.87%)	3, 990 (51.36%)	4, 279 (31.89%)
Surgery/autopsy	19 (1.02%)	17 (0.60%)	3 (1.20%)	2 (0.51%)	9 (2.93%)	86 (1.11%)	136 (1.01%)
Biochemistry/immunology	13 (0.70%)	33 (1.17%)	0 (0)	3 (0.77%)	5 (1.63%)	812 (10.45%)	866 (6.45%)
Biopsy/cytology	1, 729 (92.61%)	2, 640 (93.25%)	242 (96.41%)	364 (92.86)	224 (72.96%)	1, 118 (14.39%)	6, 317 (47.08%)
Total	1, 867	2, 831	251	392	307	7, 769	13, 417
^*^The numbers of cases and their percentages by column were presented above.							

### 2.4 生存分析

用于生存分析的数据中，完全数据为9, 705例，占72.33%，截尾数据3, 712例，占27.67%。2002年-2009年浦东新区肺癌发病患者中位生存期为280.81天。明确组织学分型的肺癌患者中位生存期为410.72天，1年-5年生存率分别为35.98%、28.51%、24.26%、21.53%、18.11%，见表 3。不同组织学类型肺癌患者生存率差异有统计学意义（*χ*^2^=23.10, *P* < 0.001），其中，腺鳞癌患者生存情况较好，小细胞癌最差，见图 2。

**3:** 2002年-2009年浦东新区不同组织学类型肺癌患者生存率 Survival time and rate of lung cancer by different histologies in Pudong New Area, 2002-2009

Histologies	Median survival time (d)	Survival rate		
		1-year	3-year	5-year
Squamous cell carcinoma	411.72	37.36%	25.06%	17.11%
Adenocarcinoma	425.71	35.97%	24.55%	19.07%
Adenosquamous carcinoma	557.62	44.99%	28.40%	26.78%
Small cell carcinoma	332.77	27.14%	16.84%	11.17%
Other specific lung cancer	344.76	31.24%	22.46%	18.66%
Total (no classification excluded)	410.72	35.98%	24.26%	18.11%
Total (no classification include)	280.81	24.27%	14.47%	9.84%

**2:**
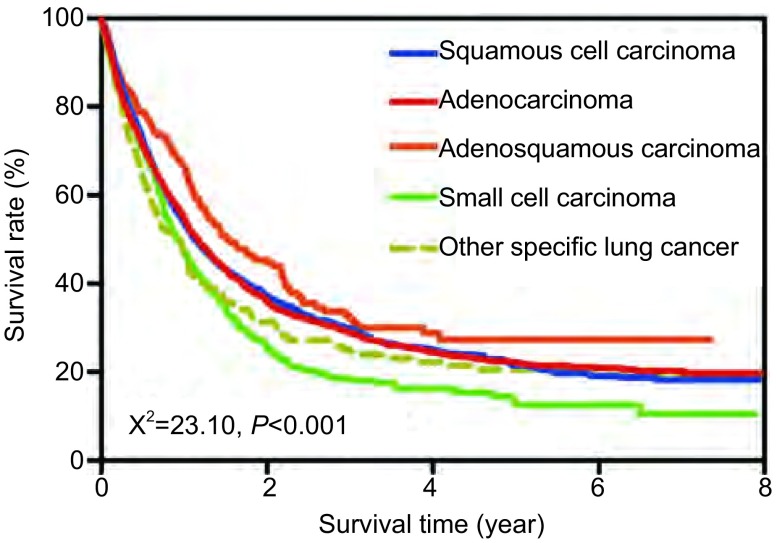
2002年-2009年上海市浦东新区居民不同组织学类型肺癌生存情况。不同组织学类型肺癌患者生存率差异有统计学意义（*χ*^2^=23.10，*P* < 0.001）。 Survival function diagram for patients died from lung cancer with different histologies in Pudong New Area, Shanghai, 2002-2009. Statistical significance was found among lung cancer with different histologies, and the chi square value was 23.10.

### 2.5 不同组织学类型生存期影响因素

结果显示，原浦东地区男性，特别是35岁-59岁，其肺癌组织学类型对生存期的影响有统计学意义（*P* < 0.01），其中腺鳞癌生存情况较好，小细胞癌最差。而原南汇地区男性鳞癌生存情况较好，小细胞癌和其他明确分型肺癌生存情况差（*P* < 0.001）。原南汇地区女性其他明确分型肺癌生存情况差（*P* < 0.01），见表 4。

**4:** 2002年-2009年浦东新区肺癌不同组织学类型生存情况 Survival condition for patients died from lung cancer with different histologies in Pudong New Area, Shanghai, 2002-2009

	Former Pudong Area			Former Nanhui Area	
	Chi square value^*^	*P*		Chi square value^*^	*P*
Gender					
Male	16.73	0.002		33.84	< 0.001
Female	7.47	0.113		6.78	0.148
Age (year)					
< 35	1.92	0.590		0.17	0.683
35-59	22.61	< 0.001		6.67	0.155
60-69	1.30	0.862		8.41	0.078
70-79	4.52	0.340		18.08	0.001
> 80	4.92	0.295		1.47	0.832
^*^The chi square values represent statistical difference among different histologies.					

## 3 讨论

本研究结果显示，2002年-2009年上海市浦东新区男、女肺癌标化发病率高于同期世界发达国家水平（男性ASR为47.4/10万；女性ASR为18.6/10万）。不同组织学类型肺癌发病率及发病趋势并不相同，不同性别各组织学类型发病趋势也不同。性别、年龄、居住地对不同组织学类型肺癌患者生存期有影响。

本研究发现在明确组织学分型的肺癌患者中，腺癌已超过鳞癌成为最主要的肺癌组织学类型，且男女肺腺癌发病率均呈上升趋势，这与国外许多研究结果一致^[7-10]^。既往有研究^[11-13]^显示，肺腺癌可能与家族肿瘤史、烹调油烟、维生素摄入量等因素有关。新近一项研究^[14]^发现，一氧化氮气体与肺腺癌有剂量-反应关系，此外，泥土中重金属污染也被认为与肺腺癌的发病有关^[15]^。

男性依然是肺癌发病的主要人群，这与男性普遍吸烟，环境烟草高暴露有关^[16]^。通常认为，肺鳞癌、小细胞肺癌与吸烟关系密切^[17]^。本次研究发现，男性小细胞癌粗发病率呈上升趋势，但男性鳞癌发病率尚没有明显变化。而在某些开展控烟效果较好的国家，已经发现男性肺鳞癌发病率出现下降趋势^[18]^。值得注意的是，本研究发现女性鳞癌粗发病率呈上升趋势。我国是一个燃煤大国，最新研究^[19]^显示，室内焚燃产生的环境纳米颗粒物是非焚燃时的16倍。研究^[20, 21]^表明，室内燃煤是增加我国女性，特别是不吸烟女性罹患肺癌风险的危险因素。此外，我国女性接受吸烟的时间较男性晚30年左右^[22]^，有高吸烟特点的女性尚未达到肺癌高风险的年龄，不断上升的女性肺鳞癌患者提示未来女性肺癌的发病率和死亡率可能会进一步增高^[23]^。因此，女性肺癌防治工作将是下一阶段肺癌防治的重要内容之一。

病理组织学诊断的比例是评价恶性肿瘤登记质量的最重要指标之一，显示了被登记的恶性肿瘤病例的可靠性。然而，肺癌的病理学诊断比例仍不足50%。这一方面由于肺癌发病集中在60岁以上老年人群^[24, 25]^，其体质相对较弱，而癌症作为消耗性疾病，容易引起老年肺癌患者身体疲乏、食欲差，从而加剧病情，影响手术及术后病理诊断。另一方面，提示晚期肺癌病例仍占很大比例，难以手术治疗，影响组织学分型。为此，提高肺癌早发现、早诊断水平，有效饮食指导改善肺癌患者生存状况，有助于延长肺癌患者生存期，对于提高肺癌患者生活质量具有重要的现实意义^[26-28]^。

本次研究纳入人群来自浦东新区，由原浦东地区和原南汇地区构成：前者城市化明显，经济条件更好，后者则仍保留大量农村地区，经济相对落后。分居住地比较不同性别、年龄的肺癌组织学类型对患者生存期的影响结果发现，原南汇地区男性鳞癌生存情况较好，可能与其鳞癌发病诊断年龄较早有关。原南汇地区其他明确分型肺癌的生存情况较差，可能与原浦东地区经济条件较好有关，提示城乡医疗水平以及卫生服务资源差别可能影响肺癌患者生存率^[29]^。

综上所述，上海市浦东新区肺癌发病水平仍然很高，其中肺腺癌发病率最高并呈上升趋势，女性肺癌发病率有上升趋势。不同组织学类型肺癌患者生存期不同，并受性别、年龄、居住地等因素影响。随着我国进一步老龄化，在未来一段时间里，肺癌防控仍将是我国公共卫生领域的重点课题。
